# The effect of short-term consumption of *Bifidobacterium bifidum* on the gut microbiome of obese individuals

**DOI:** 10.3389/ebm.2026.10894

**Published:** 2026-02-23

**Authors:** Inna Burakova, Yuliya Smirnova, Polina Morozova, Svetlana Pogorelova, Olga Kryukova, Tatiana Kislova, Olga Korneeva, Mikhail Syromyatnikov

**Affiliations:** 1 Laboratory of Metagenomics and Food Biotechnology, Voronezh State University of Engineering Technology, Voronezh, Russia; 2 Department of Physical Culture and Sports, Voronezh State University of Engineering Technologies, Voronezh, Russia

**Keywords:** *Bifidobacterium bifidum*, high-throughput sequencing, microbiome, obesity, probiotics

## Abstract

It is known that gut microbiota dysbiosis can lead to obesity by disrupting energy consumption and metabolism. Probiotic supplements are a potential therapeutic option for improving intestinal homeostasis. The aim of this study was to investigate the effect of a probiotic supplement containing *Bifidobacterium bifidum* on the intestinal microbiome of people with obesity using high-throughput sequencing on the DNBSEQ-G50 platform. The study demonstrated a positive effect of the supplement on bacterial species such as *Bacteroides uniformis*, *Alistipes putredinis*, *Alistipes shahii*, *Dysosmobacter welbionis*, and *Gemmiger formicilis*. Therefore, we suggest the potential use of this bacterial species in the treatment of gut microbiota dysbiosis of obese individuals.

## Impact statement

Why is the work submitted important to the field? This work addresses a gap in understanding how specific probiotic interventions can therapeutically modulate gut dysbiosis in obesity. Since obesity-related metabolic disorders are increasingly recognized as having a microbial component, demonstrating that a single probiotic strain can predictably shift the microbial community toward beneficial species provides a foundation for targeted therapeutic approaches rather than broad, non-specific interventions. How does the work submitted advance the field? The study advances from observational correlations between microbiome composition and obesity to demonstrating causal intervention effects. By using high-throughput sequencing to track species-level changes, it moves beyond genus-level descriptions that dominated earlier probiotic research. The identification of specific bacterial species that respond to Bifidobacterium bifidum supplementation creates a measurable framework for evaluating probiotic efficacy and establishes biomarkers for treatment response. What new information does this work impart to the field? The work provides concrete evidence that Bifidobacterium bifidum supplementation promotes four specific beneficial species while suppressing a metabolically unfavorable one. This species-level resolution reveals the ecological dynamics of probiotic action—showing that probiotics don't simply colonize but rather reshape the existing community structure. The observed increase in alpha diversity suggests that the probiotic acts as an “ecological” engineer, creating conditions that support broader microbial diversity rather than simply displacing existing species. How does this new information impact the field? This information enables more rational probiotic selection for obesity management and provides mechanistic insight into how probiotics exert metabolic benefits. Researchers can now design studies targeting these specific microbial shifts, develop diagnostic tools to identify patients most likely to respond, and potentially combine probiotics with other interventions that support these beneficial species. It transforms probiotic therapy from empirical treatment to precision intervention.

## Introduction

In recent years, one of the significant problems for humanity in developed countries is obesity, which can increase the risk of developing hypertension, diabetes, and cardiovascular diseases [1]. Moreover, a significant portion of previous studies have shown that people diagnosed with obesity have changes in the composition of their intestinal microbiota [2]. However, solving this problem is complicated by the fact that obesity has a complex etiology, including both genetic and environmental factors [3].

One treatment option is the use of probiotics to alter the composition of the intestinal microbiota [4]. In turn, probiotics, due to their ability to regulate intestinal microbiota, can have a beneficial effect on the intestines of people who are overweight or obese, but the mechanisms of their action are not fully understood and require further research [5].

In the human body, *Bifidobacterium* is one of the key intestinal commensals capable of providing various health benefits; due to these properties, these bacteria are widely used as probiotics [6].

The beneficial role of this bacterial species is supported by studies using a supplement containing *Bifidobacterium longum* to protect the host from metabolic syndromes, including diet-induced obesity [7]. This is supported by studies noting that the bacterial species *Bifidobacterium* is significantly lower in overweight/obese children compared to normal weight children, suggesting that it is involved in fat accumulation and obesity [8].

Therefore, the aim of the study was to evaluate the effect of a probiotic supplement containing *Bifidobacterium bifidum* on the gut of people diagnosed with obesity.

## Materials and methods

### Object of study

At first, patients completed a questionnaire that included the following questions: age, diet, physical activity, history of infectious and non-infectious diseases, medication use, including hormonal and antibacterial medications (over the past 6 months), anthropometric parameters [height, weight for calculating body mass index (BMI)] and waist circumference (WC). Based on the questionnaire results, 14 patients with a BMI greater than 30 were selected. The average age of the group was 38 years.

During the first phase of the study, stool samples (at least 1 g) were collected from patients included in the study group. Then, without changing their usual diet, they took a probiotic supplement containing *B. bifidum* VSUET22 (5 × 10^8^ CFU) twice daily with food for 2 weeks. After completing the 2-week supplementation period, stool samples were collected for further molecular genetic testing.

### DNA extraction

Total DNA was isolated from each fecal sample obtained using a commercial HiPure Microbiome DNA Kit (Magen, China).

Total DNA quantification was performed using a Qubit 2.0 Fluorometer (Thermo Fisher, USA) and a QuDye® HS Double-Stranded DNA Assay Kit (Lumiprobe, Russia). Purity and impurity assessment were performed using a Nano-500 Spectrophotometer (Allsheng, China) at 230, 260, and 280 nm, applying 2 µL of the resulting DNA sample to the detector.

All manipulations were carried out according to the protocols of the manufacturers of commercial kits.

### Library preparation and sequencing on the DNBSEQ-G50 platform

The commercial MGIEasy Fast FS DNA Library Prep Set User Manual and UDB Primer Adapter Kit were used for library preparation. Circularization of single-stranded DNA was performed using the MGIEasy Dual Barcode Circularization Module (MGI, China). Subsequent DNA generation and cartridge loading were performed using the FCL PE100/FCS PE 150 DNBSEQ-G50RS High-throughput Sequencing Kit (MGI, China). Further sequencing was performed using the FCL DNBSEQ-G50RS Sequencing Flow Cell. All manipulations were performed according to the manufacturer’s protocols.

### Bioinformatic and statistical data analysis for the DNBSEQ-G50 platform

Raw metagenomic read quality was evaluated with FastQC (v0.12.1). Adapter and other technical sequences were removed using flexbar (v3.5.0). To deplete host-derived reads, sequences were mapped with Bowtie2 against the human (GCF_000001405.40) reference genomes, and matching reads were discarded. Taxonomic composition was inferred with MetaPhlAn 4 (v4.1.1) using the default bacterial, viral, and eukaryotic marker databases. Microbial functional potential and pathway abundances were profiled with HUMAnN. Antibiotic resistance gene content was characterized with GROOT using a ARG-ANNOT index.

### Statistics

Statistical analysis was performed in R (Version 4.5.1). Alpha diversity was assessed using the Shannon index, followed by the nonparametric Mann-Whitney test. Beta diversity was analyzed using the Bray-Curtis distance metric, followed by the ADONIS function. For a more detailed analysis of pairwise comparisons between groups, PERMANOVA was used. Differences in the relative abundance of bacterial species between groups were analyzed using the ANCOMBC method (Version 2.11.1).

Results were considered statistically significant at an adjusted p-value ≤0.05. Data are presented as mean ± standard deviation (SD).

## Results

Analysis of the fecal microbiome of patients before and after taking *B. bifidum* identified 8 phyla, 26 classes, 29 orders, 41 families, 107 genera, and 170 species of bacteria.

**FIGURE 1:**
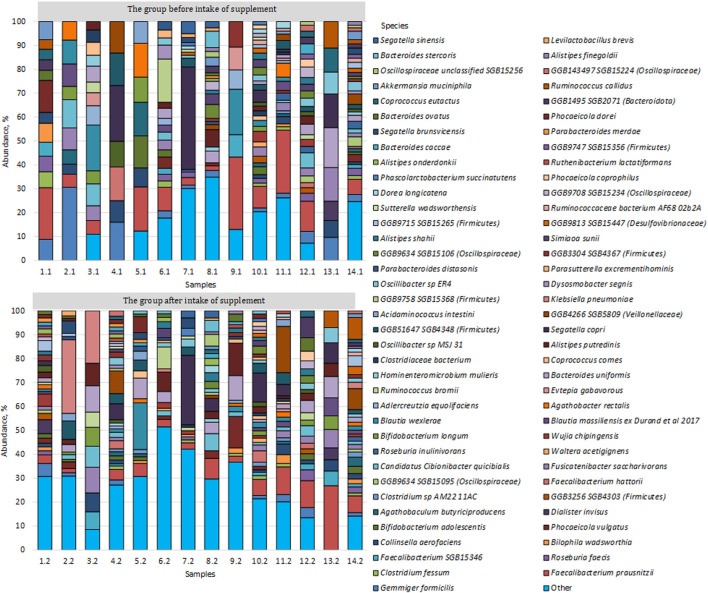
Bacterial species found in the microbiome of the study groups.

We conducted a comparative analysis of the microbiome of the study groups, during which we identified 73 of the most common species, the number of which exceeded 1%, all other species were grouped as “Others” (Figure 1).

**FIGURE 2:**
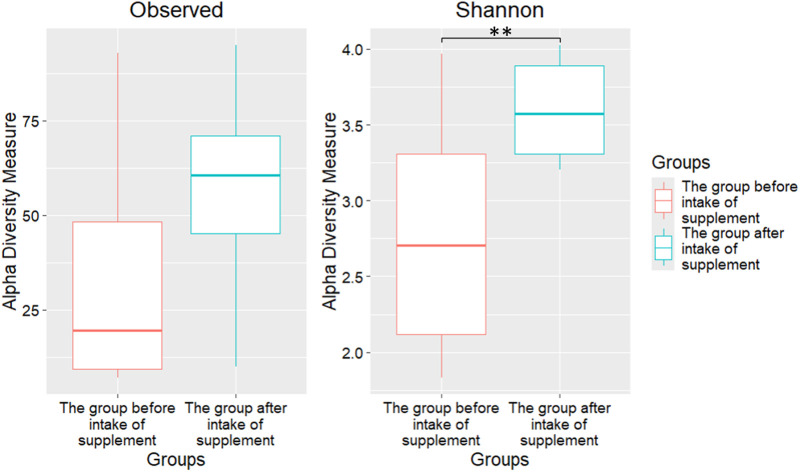
Alpha diversity of the intestinal microbiome of patients before and after taking *Bifidobacterium bifidum*. **p ≤ 0.01.

An alpha diversity analysis was also performed using measures of observed species diversity and the Shannon index (Figure 2). Statistically significant differences in the Shannon index (2.77 ± 0.73 vs. 3.60 ± 0.29, p = 0.007) were found between the gut microbiomes of patients before and after *Bifidobacterium bifidum* administration, respectively.

**FIGURE 3:**
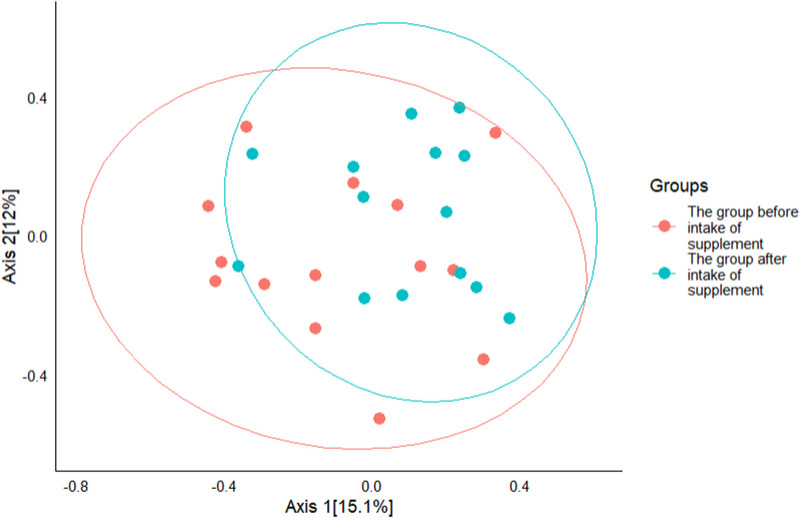
Principal coordinates analysis (PCoA) plot of beta diversity based on the Bray-Curtis scale between study groups.

Beta diversity analysis also showed the presence of clustering between the studied groups, however, no statistically significant differences were found p = 0.166 (Figure 3).

**FIGURE 4:**
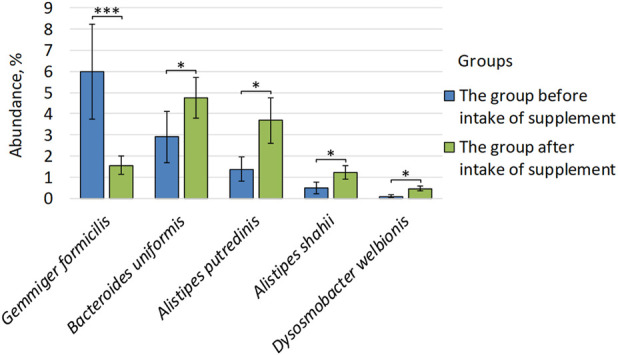
Differences in the composition of the intestinal microbiome of patients before and after taking *Bifidobacterium bifidum*. *p ≤ 0.05, ***p ≤ 0.001.

Differential abundance analysis revealed statistically significant differences at the species level between the microbiome of patients before and after taking *Bifidobacterium bifidum*. Thus, in patients after taking the supplement, we observed an increase in the number of species: *Bacteroides uniformis* (2.91% ± 1.23 vs. 4.76% ± 0.96, p = 0.03), *Alistipes putredinis* (1.39% ± 0.57 vs. 3.68% ± 1.08, p = 0.04), *Alistipes shahii* (0.51% ± 0.28 vs. 1.24% ± 0.32, p = 0.04), *Dysosmobacter welbionis* (0.09% ± 0.06 vs. 0.47% ± 0.13, p = 0.01). In contrast, the relative abundance of *Gemmiger formicilis* was reduced by probiotic supplementation compared to pre-supplementation levels (5.98% ± 2.25 vs. 1.57% ± 0.44, p = 4.42E-06) (Figure 4).

## Discussion

After consumption of *Bifidobacterium bifidum*, an increase in the relative content of *Bacteroides uniformis*, *Alistipes putredinis*, *Alistipes shahii*, *Dysosmobacter welbionis* was observed. An increase in the relative amount of *Gemmiger formicilis* was also observed. Changes toward an increase in bacteria producing short-chain fatty acids (SCFAs), such as acetate, propionate, and butyrate, are of particular interest in the context of metabolic health. SCFAs are key mediators of microbiota-host interactions: they serve as a major energy source for colonocytes, strengthen the intestinal barrier, have anti-inflammatory properties, and regulate energy homeostasis [9]. Thus, the observed shift may be one of the mechanisms by which the probiotic exerts a potentially beneficial effect in obesity.

One possible explanation is that bifidobacteria ferment carbohydrates into short-chain fatty acids. These metabolites can then be utilized as substrates by *Bacteroides* and *Alistipes*, which are able to metabolize them. The bacterial species *G. formicilis* is known to produce butyric acid, a short-chain fatty acid, in the body and has also been shown to be reduced in abundance in inflammatory bowel disease [10, 11]. However, the abundance of the bacterial species *G. formicilis* is associated with a higher risk of developing colitis [12]. In turn, a study of patients with colorectal cancer showed a decrease in this bacterial species in the group of patients with a positive test for the presence of blood in the stool [13]. Also, in a study of type 2 diabetes mellitus, an increase in the number of this species relative to the control group was recorded after 12 weeks of therapy [14]. *Gemmiger formicilis* was also found to be abundant in a group of patients with chronic heart disease who suffered from headache due to excessive use of nonsteroidal anti-inflammatory drugs, relative to the control group [15]. Our study showed a decrease in the abundance of this bacterial species when taking a probiotic supplement containing *B. bifidum*. Thus, it is difficult to unambiguously interpret the influence of bacteria of this species on the development of obesity.

*Bacteroides uniformis* is considered a beneficial species for the host organism, as it is capable of producing short-chain fatty acids and other metabolites [16]. *B. uniformis* abundance was shown to be reduced in metabolically associated fatty liver disease and was also negatively correlated with hepatosteatosis and BMI [17]. It was also shown that the abundance of this bacterial species was significantly higher in healthy controls compared to patients with ulcerative colitis [18]. Previously, the possibility of using representatives of this bacterial species as a new probiotic bacterium in the treatment of colon diseases associated with dysbacteriosis was proposed [18]. Our study also observed an increase in *B. uniformis* abundance in a group of patients after taking a probiotic supplement containing *B. bifidum*, which is consistent with other studies.

The genus *Alistipes* is often associated with anti-inflammatory effects in certain cardiovascular diseases, colitis, liver fibrosis, and cancer immunotherapy. However, there is also evidence suggesting pathogenicity in colorectal cancer and its association with mental disorders [19]. The bacterial species *A. putredinis* is a potentially beneficial member of the gut microbiota associated with metabolic health [20, 21]. An increase in this bacterial species has been shown after mucosal healing in ulcerative colitis, as well as the contribution of *A. putredinis* to amino acid metabolic pathways [22]. However, at the same time, a link was found between increased transcriptional activity of *A. putredinis* and increased severity of Crohn’s disease [23]. An increase in the number of this bacterial species was observed after taking a probiotic supplement containing *B. bifidum*. The dual role of this type of bacteria in the development of various diseases does not allow us to draw a clear conclusion regarding the influence of *A. putredinis* on the pathogenesis of obesity.

Representatives of the species *A. shahii* have a negative correlation with the inflammatory process [24]. The increase in the number of *A. shahii* is associated with a positive effect on the state of the intestine: improvement of the epithelial barrier and changes in metabolites in feces [25]. Low abundance of this species is observed in ulcerative colitis and Crohn’s disease [26]. Our study found an increase in *A. shahii* abundance after probiotic supplementation. Thus, it can be assumed that the intake of probiotics had a positive effect on the number of beneficial bacteria of the *A. shahii* species.

*Dysosmobacter welbionis* is a recently described commensal bacteria being studied as a promising new probiotic due to its ability to produce butyrate. In a mouse study, oral administration of live *Dysosmobacter welbionis* J115T partially prevented the development of insulin resistance and inflammation in white and brown adipose tissue. The protective effect was associated with an increase in mitochondria and activation of nonshivering thermogenesis in brown adipose tissue [27]. Low abundance of *D. welbionis* is often associated with pathological conditions such as obesity and diabetes [28]. Our study showed that taking a probiotic supplement containing *B. bifidum* was associated with a significant increase in this bacterial species. This is consistent with literature data and suggests that taking this probiotic promotes normalization of the intestinal microbiota.

A critical limitation of short-term probiotic use is the stability and prolongation of microbiota restructuring. The presence of probiotic microorganisms in the intestine can often be transient. Their numbers fluctuate depending on whether or not they are discontinued, and the microbiota often strives to return to its usual homeostasis. A study [29] demonstrates the effect of probiotics on microbiota restoration and emphasizes that this effect may be context-dependent and does not necessarily lead to long-term community restructuring after completion of the course. The ability of probiotic strains to establish themselves is also associated with the initial microbiota [30], which limits the possibility of long-term colonization with short courses of probiotic use. Therefore, further research is needed to show changes in the intestinal microbiota of people with obesity over a long period of time after consumption of *B. bifidum*.

Our results suggest that the *B. bifidum* strain has the potential to correct dysbiotic disturbances in the microbial community in obesity. A change in microbial composition toward an increased proportion of beneficial commensals may be one of the mechanisms underlying the beneficial effects of probiotics on metabolism.

## Data Availability

The datasets presented in this study can be found in online repositories. The names of the repository/repositories and accession number(s) can be found below: https://www.ncbi.nlm.nih.gov/bioproject/?term=PRJNA1353102, NCBI BioProject PRJNA1353102.
